# Method: automatic segmentation of mitochondria utilizing patch classification, contour pair classification, and automatically seeded level sets

**DOI:** 10.1186/1471-2105-13-29

**Published:** 2012-02-09

**Authors:** Richard J Giuly, Maryann E Martone, Mark H Ellisman

**Affiliations:** 1 Center for Research in Biological Systems, University of California, 9500 Gilman Dr., La Jolla, CA 92093 USA

## Abstract

**Background:**

While progress has been made to develop automatic segmentation techniques for mitochondria, there remains a need for more accurate and robust techniques to delineate mitochondria in serial blockface scanning electron microscopic data. Previously developed texture based methods are limited for solving this problem because texture alone is often not sufficient to identify mitochondria. This paper presents a new three-step method, the Cytoseg process, for automated segmentation of mitochondria contained in 3D electron microscopic volumes generated through serial block face scanning electron microscopic imaging. The method consists of three steps. The first is a random forest patch classification step operating directly on 2D image patches. The second step consists of contour-pair classification. At the final step, we introduce a method to automatically seed a level set operation with output from previous steps.

**Results:**

We report accuracy of the Cytoseg process on three types of tissue and compare it to a previous method based on Radon-Like Features. At step 1, we show that the patch classifier identifies mitochondria texture but creates many false positive pixels. At step 2, our contour processing step produces contours and then filters them with a second classification step, helping to improve overall accuracy. We show that our final level set operation, which is automatically seeded with output from previous steps, helps to smooth the results. Overall, our results show that use of contour pair classification and level set operations improve segmentation accuracy beyond patch classification alone. We show that the Cytoseg process performs well compared to another modern technique based on Radon-Like Features.

**Conclusions:**

We demonstrated that texture based methods for mitochondria segmentation can be enhanced with multiple steps that form an image processing pipeline. While we used a random-forest based patch classifier to recognize texture, it would be possible to replace this with other texture identifiers, and we plan to explore this in future work.

## Background

The improved resolution and amount of detail afforded by emerging electron microscopy techniques, such as serial block-face scanning electron microscopy (SBFSEM) [1], is enabling researchers to explore scientific questions that were previously impossible. SBFSEM enables mapping of subcellular structures within large 3D regions, 1 mm × 2 mm in the XY plane and greater than 0.5 mm in Z. However, the interpretation of data acquired with these techniques requires high-throughput segmentation that addresses the complexity and multi-scale nature of these data.

### Biological motivation

**Figure 1:**
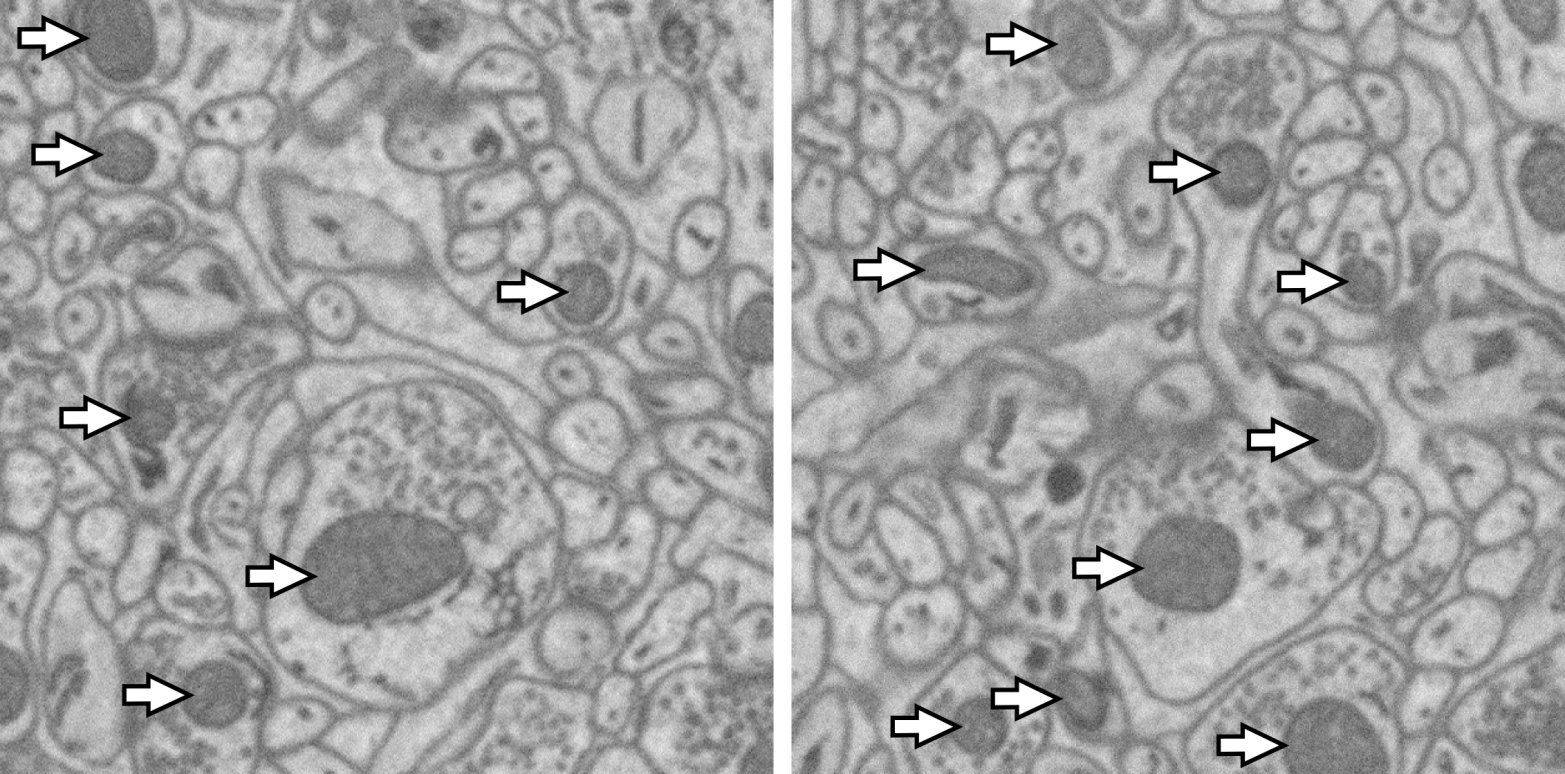
**Examples of mitochondria in SBFSEM micrographs of mouse cerebellum**. Arrows indicate mitochondria. Images are 3.1 μm × 3.1 μm.

The morphology and distribution of mitochondria has biological significance. For example, morphology of mitochondria has been studied as a means to detect abnormal cell states such as cancer [2]. Additionally, abnormal morphologies and distributions of mitochondria are associated with neural dysfunction and neurodegenerative disease [3]. As described previously, SBFSEM techniques, coupled to new staining protocols [4], are able to reveal both cell boundaries and many membrane-bounded intracellular components, such as mitochondria. Figure 1 shows slices of mouse cerebellum from a volume acquired with a specialized scanning electron microscope equipped with a high precision Gatan 3View ultramicrotome for serial blockface imaging, which involves use of a vibrating diamond knife to precisely plane away material from the surface of a specimen while imaging.

Current methods for extracting information from complex cellular datasets reflect a long history of incremental development. Following specimen preparation and data acquisition, image stacks must be segmented before cellular structure-function relationships can be fully analyzed. During segmentation, compartments of interest are delimited. Since segmentation is typically performed by hand or semi-automatically with manual correction, it can be notoriously time consuming and represents a clear bottleneck in cellular imaging [5,6]. In a typical scenario, segmentation involves a single trained expert using automated algorithms or manually going through each individual slice and tracing contours around the structures of interest using a program such as IMOD [7], JINX [8], or any number of other specialized programs.

### Serial blockface imaging modality

Specifically, this paper addresses segmentation of mitochondria in SBFSEM data. Other previously addressed technologies are serial section electron microscopy and focused ion beam serial electron microscopy (FIBSEM). We chose to use SBFSEM because it achieves full automation, acquires rapidly, produces well registered images, and has commercial availability. While FIBSEM has ability to image with higher Z resolution (5-6 nm between slices), SBFSEM affords a larger imaging surface and higher speed. Use of a microtome with SBFSEM is faster than the ion milling process of FIBSEM and allows for a larger cutting surface, ~2 mm^2^, compared to ~0.5 mm^2 ^for FIBSEM[9].

Ability to rapidly scan tissue is important when acquiring large datasets and studying the distribution of structures within tissue. Acquisition time increases with finer resolution in XY and also increases with smaller Z step size [1]. While sampling with larger steps in X, Y, and Z requires that the automatic segmentation operate on sparser data, it makes the image acquisition more practical in terms of time and disk storage. We chose an XY pixel size of 10 nm × 10 nm and Z step size of 50 to 70 nm. At a rate of 10 microseconds per voxel, this would allow an acquisition rate of 0.5 to 0.7 cubic microns per second. Because a full dataset can cover millions of cubic microns, imaging can require multiple days of acquisition time and the acquisition rate is critical.

For each test, two subsets of data used for testing our method in this paper has dimensions of 3.5 microns × 3.5 voxels × 0.75 microns. We chose 10 nm × 10 nm × 50-70 nm voxel size suitable for imaging large blocks in reasonable time, and we show that our method is robust enough to perform well even with anisotropic resolution and sparse sampling (especially in the Z direction). The purpose of this work is to demonstrate accuracy and time feasibility of this method on test samples. We used a single core processor for all of the testing reported here. As future work, we plan to process full datasets using parallel processing resources with thousands of cores.

### Previous work in automatic segmentation

The electron microscopic staining and imaging technology used for this work highlights intracellular structures, such as vesicles and mitochondria, as well as cellular membranes resulting in complex, textured images. While staining of multiple structures makes it possible to accomplish the identification of most cellular and subcellular tissue components simultaneously, it makes automatic segmentation and identification of these more challenging. Automatic segmentation accuracy is critical, as each manual correction requires human effort and ultimately increases the time and cost required for segmentation. Modern three dimensional TEM and SEM images involve a large number of objects with various three dimensional shapes. Image intensity alone does not accurately identify a given structure, and identification of objects typically involves a knowledge of various textures and shapes present in the data. Therefore, the numerous segmentation algorithms developed for other biomedical imaging modalities are not directly applicable to thin sections from TEM and serial block face derived SEM images. Level-set [10] and active contour segmentation methods are not effective when *directly *applied to automatically segmenting mitochondria in the data presented here because the edge attraction terms used in these methods are easily confused by the presence of significant textures. (However, we establish that a level set may be used effectively as a final step in a process.)

While progress has been made to develop automatic segmentation techniques appropriate for mitochondria [2,11-15] and cells [16-20], there remains a need for more accurate, rapid, and robust techniques to delineate mitochondria in SBFSEM data. In [14,2], and [15], primarily texture detection is used to segment mitochondria. Although texture based methods may be appropriate for high resolution thin section transmission electron microscopy (TEM) images, current SBFSEM technology does not provide the resolution required for distinct textures in neuropil.

In typical SBFSEM data, separation between XY planes may be greater than FIBSEM, with typical ranges of 30-100 nm, giving lower effective resolution in Z than × and Y. Rather than using only 3D operations, we use a combination of 2D and 3D processing. This allows us to take advantage of the higher resolution available in the XY plane. A 2D patch classifier is used at step 1. For step 2, we use a custom method of 2D contour identification which is based on isocontour detection and contour pair filtering. Use of 2D contours is advantageous because the contours often outline the mitochondria, which have various but often recognizable shape. At step 3, we use a 3D level set operation which increases the 3D smoothness of the detected structure and helps increase the true positive rate.

Lucchi et al. [12,13] published a method for mitochondria segmentation in FIBSEM images achieving pixel classification accuracy as high as 98%. Note that accuracy is defined on pixel classification as (TP + TN)/(TP + FP + FN + TN), where TP is the number of true positives, TN is the number of true negative, FP is the number of false positives, and FN is the number of false negatives. To utilize 3D image information, Lucchi et al. used a classifier to recognize which pairs of 3D supervoxels are most likely to straddle a relevant object boundary. In their FIBSEM data, X, Y, and Z resolution are all 5-6 nm, which makes use of 3D supervoxel approach appropriate. However, to address SBFSEM data with generally coarser and anisotropic resolution, we use a different approach as described above.

In other previous work [21], shape rather than texture information is used for *detection *of mitochondria in 2D slices of FIBSEM data. Our goal differs from this in that we are concerned with segmentation, not only detection, in 3D images, and our approach uses information in more than one plane to discriminate between mitochondria and other objects.

We have previously explored use of texture and shape to automatically segment mitochondria in tomography [22], and SBFSEM [23]. In this work we present our complete multi-step method and test it against human segmentation. We demonstrate that similar accuracy can be achieved with SBFSEM which has different resolution characteristics than aforementioned techniques but allows rapid automated 3D scanning of relatively large three dimensional regions with commercially available microscopes.

## Methods

**Figure 2:**
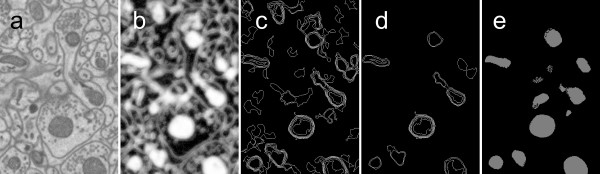
**Automatic results for mitochondria segmentation in SBFSEM images**. XY slices shown here are 2.1 μm × 3.1 μm. (a) Original data for Test 1. (b) Step 1: Voxel classification result and contour detection. (c) Step 2: Detection of isocontours. (d) Salient contours after contour classification. Notice that many contours that do not belong to mitochondria have been eliminated. (e) Step 3: Result of level set operation seeded with inner region of salient contours. Note that the small isolated sets of pixels in (e) are a result of the 3D level set operation spreading into a few pixels when seeded from an adjacent plane. Also, observe that contours occasionally are eliminated in the contour pair classification set but re-appear after the level set operation. This is the result of contour classification eliminating a contour in one plane but accepting contours in nearby planes above or below. Then salient contours above and below act as seed point regions, and the 3D level set operation tends to fill in the region where the contour was eliminated.

The Cytoseg process (CP) for segmentation presented here consists of three steps (see Figure 2): patch classification, contour set classification, and a level set operation. Each step produces output that feeds the following step(s) of the process. For the first step, we use a random forest classification technique for identification of pixels that belong to subcellular objects. This step serves to identify texture. The input vector is a set of values from an N×N patch of pixels. Results with mitochondria segmentation indicate that the patch classification learning process can be accomplished rapidly (for 1591 image patches, 13 seconds on single 2.6 GHz processor). For the second step, we perform random-forest-based classification on contours or pairs of contours generated by outlining connected components after thresholding the patch classification output with multiple thresholds. To produce smooth 3D objects, we apply a third step in which contours are used to find seed points for the initialization of geodesic active contour operations (from the Insight Segmentation and Registration Toolkit (ITK)) [24], which produces 3D blobs in an array of voxels. The following describes each step in detail:

### Step 1: 2D patch classification

The first step consists of a patch classifier using a random forest [25]. A training data set is used with each pixel labeled mitochondria or not. The classifier uses raw pixel values from 2D image patches. The output of step 1 is a map of mitochondria probability. We chose 2D rather than 3D patches to produce a method that would be robust when there are significant gaps between XY slices.

### Step 2: Contour Set Classification

In the second step, contours in XY planes of the volume are detected using multiple isocontours applied to the probability map from the pixel classifier of step 1. Isocontours are detected at 12%, 26%, 40%, 54%, 68%, 82%, and 96% of the total intensity in the probability map (14% intervals). After detection, contour pair classification serves to eliminate the extraneous contours that do not belong to mitochondria. In this paper, C_i, j _is the contour of in XY plane i with index j. Contours of extreme size (perimeter less than 0.6 μm or greater than 6 μm) are not used. The extreme limits were determined based on the known size of mitochondria in our data. It is unlikely that such a large or small contour would delineate a mitochondria. For classification, contours can be grouped into sets of M contours in adjacent planes. We explore use of M = 1 and M = 2. When pairs of contours are used (M = 2), we eliminate pairs whose centers differ by a large amount. That is, we use all pairs C_i, m _and C_i+1, n _where Euclidean distance (in the XY plane) from the center of C_i, m _to the center of C_i+1, n _is less than D. Although the random-forest-based classification procedure is the primary means of identifying salient pairs, this elimination procedure reduces computation time by quickly removing many invalid pairs in which the two contours are very far apart are not delineating the same mitochondria. Based on experiment with our data, we chose D = 0.4 μm for results of this paper. Contour centers were calculated as the average of all points on the circumference.

The contour sets are classified based on geometric and pixel value-based features of each contour in the set. Seven features are used: (1) contour perimeter, (2) average gray value on the contour points, (3) average gray value of the probability map inside the contour, (4) area of the contour, (5) ellipse overlap, (6) ellipse width, and (7) ellipse height. Ellipse overlap is a measure of how elliptical the contour is. To compute this value, an ellipse is first fit to the contour. Ellipse width and height are the width and height of the ellipse that best fits the contour. The fraction of overlap between the area inside the ellipse and the actual contour area provides the ellipse overlap value. When two adjacent contours (C_i, m _and C_i+1, n_) are used for each example, the full feature vector consists of (1) the seven features of each contour, (2) the distance between the two contour centers, and (3) the difference of the seven features, yielding a total of 22 features per contour pair. For example, Figure 2(c) shows all detected contours and (d) shows remaining contours after classification and elimination of all pairs below a probability threshold of T = 0.25.

### Step 3: 3D level set operation

In the third step, a "geodesic active contour" level set filter is then used to produce 3D blobs that represent mitochondria. The geodesic active contour filter takes two input images (1) the result of an input fast march filter and (2) the edge potential map. In our method, the input of the fast march filter is the probability map from step 1. For the edge potential map, we used the gradient magnitude of the probability map from step 1. The filter performs a level set operation to generate a final result. Proper seeding of the input fast march is critical to produce an accurate segmentation. The inner region of the contours detected in step 2 demarcates the initial seed points, while the gradient magnitude of the 3D probability map defines the edge potential image. The inner region of contours is found by taking the full region of the contour and eroding it by 10 pixels. The edge potential image produces a "force" so that the geodesic active contour filter operation tends to fill until it reaches the boundary of mitochondria and non-mitochondria voxels. Results are shown in Figure 2(e) and 6(b).

## Results

A total of four test runs were performed to measure the performance of the method in various types of mouse brain tissue: cerebellum, dentate gyrus, and the CA3 division of hippocampus. The first test in cerebellar tissue is described in detail and then supplementary three tests are then described briefly in the additional testing section below. With each test, 15 slices are used for training data and 15 slices are used for testing. Accuracy for each of the 4 tests is reported in Table 1.

**Table 1:** Test results

Test #	Tissue	TPR	FPR (RLF)	FPR (CP)	Accuracy (RLF)	Accuracy (CP)
1	Cerebellum	0.8	0.05	0.02	0.94	0.97
2	Cerebellum	0.9	0.11	0.03	0.89	0.96
3	Dentate Gyrus	0.84	0.29	0.02	0.72	0.97
4	CA3	0.9	0.3	0.07	0.72	0.93

Results for all tests. TPR stands for true positive rate. FPR stands for false positive rate. Accuracy was calculated by comparing against manually labeled data. TPR applies to both the RLF and CP methods. For comparison, in each test, the RLF threshold was set so that its TPR would be equal to the TPR of the Cytoseg process. Note that CP consistently gives higher accuracy and lower FPR.

### Step 1: 2D Patch Classification Results

**Figure 3:**
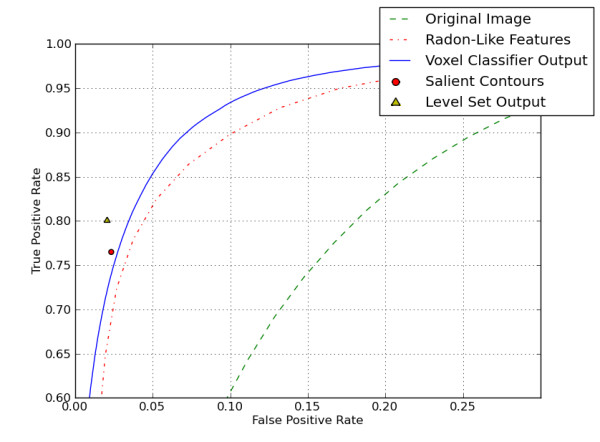
**ROC results for each step of the automatic segmentation process**. ROC results for Test 1. The dotted ROC curve is for a simple threshold of the raw data. The solid ROC curve is for the random forest based patch classifier. The dot-dash ROC curve is for the Radon-Like Features output. The circle marker shows the false positive rate and true positive rate when the pixels inside of automatically detected salient contours are marked as mitochondria (classification was performed on contour pairs). The triangle marker shows the false positive rate and true positive rate when inner points of salient contours are used to initialize a level set operation.

For Test 1, a subvolume of a 3D SBFSEM cerebellar neuropil dataset (350 voxels × 350 voxels × 30 voxels) was used. (The full volume is available at ccdb.ucsd.edu as a microscopy product with the ID 8192.) Each voxel was manually labeled mitochondria or not by an expert electron microscopist, familiar with the ultrastructural anatomy of the cellular region of the brain used to produce the test dataset. The first 15 XY slices were used to train the patch classifier, and the classifier was then run on a test set comprising the last 15 XY slices. The dimensions of the image patches were 11 × 11. An anisotropic voxel size was used, 10 nm in the XY plane with 50 nm steps in Z. A subset of pixels was selected from the training set at random to be used for training: 689 positive and 902 negative examples. The learning process required 13 seconds running on a single 2.6 GHz processor. Classification for all pixels in the test set required 80 minutes. Figure 3 shows the receiver operating characteristic (ROC) curve for this patch classification in Test 1.

Mitochondria are typically dark, and simple thresholding of pixel intensity was also tested as a baseline case for identification of mitochondria. However accuracy with thresholding alone is not sufficient, and the ROC in Figure 3 indicates that more complex methods of Radon-Like Features (RLF) and random forest classification yield more accurate results, with the random forest classification giving highest true positive rates and lowest false positive rates compared to other methods tested.

### Step 2: Contour classification results

**Figure 4:**
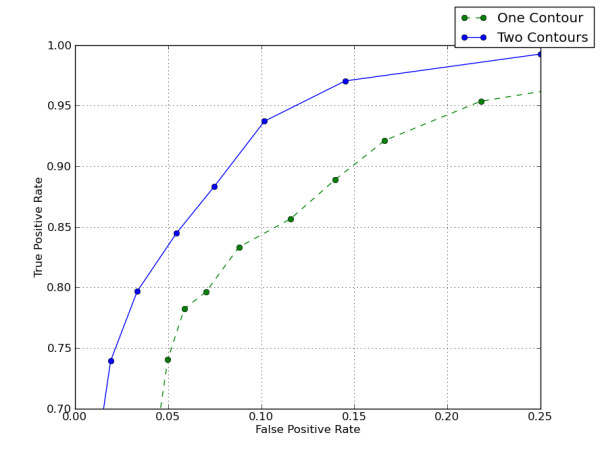
**Classification improvement when using contour pairs (M = 2) rather than single contours (M = 1)**. This compares classification of single contours to contours pairs for Test 1. (An example of salient contours in show in Figure 2 (d).) For the dotted ROC curve, each classified example is based on a single contour. For the solid ROC curve, each classified example is based on a pair of contours.

A 350 × 350 × 15 slab of data (corresponding to the output of step 1) was used for quantification of contour-set classification accuracy. To perform this test, patch classification and contour detection was first performed, as described earlier. The ground-truth labels for contours were derived from the patch classification training set: detected contours were considered salient if 90% or more of the pixels within the contour were marked as mitochondria in manually generated training data. To quantify accuracy of the contour-set classifier, 5 fold cross-validation was used. Figure 4 shows accuracy using single contours and contour pairs. There were 1340 single contours and 6175 contour pairs. Learning with single contours required 2 seconds. Classification of single contours required 1 second. Learning with contour pairs required 22 seconds. Classification of contour pairs required 5 seconds.

### Step 3: Level set results

**Figure 5:**
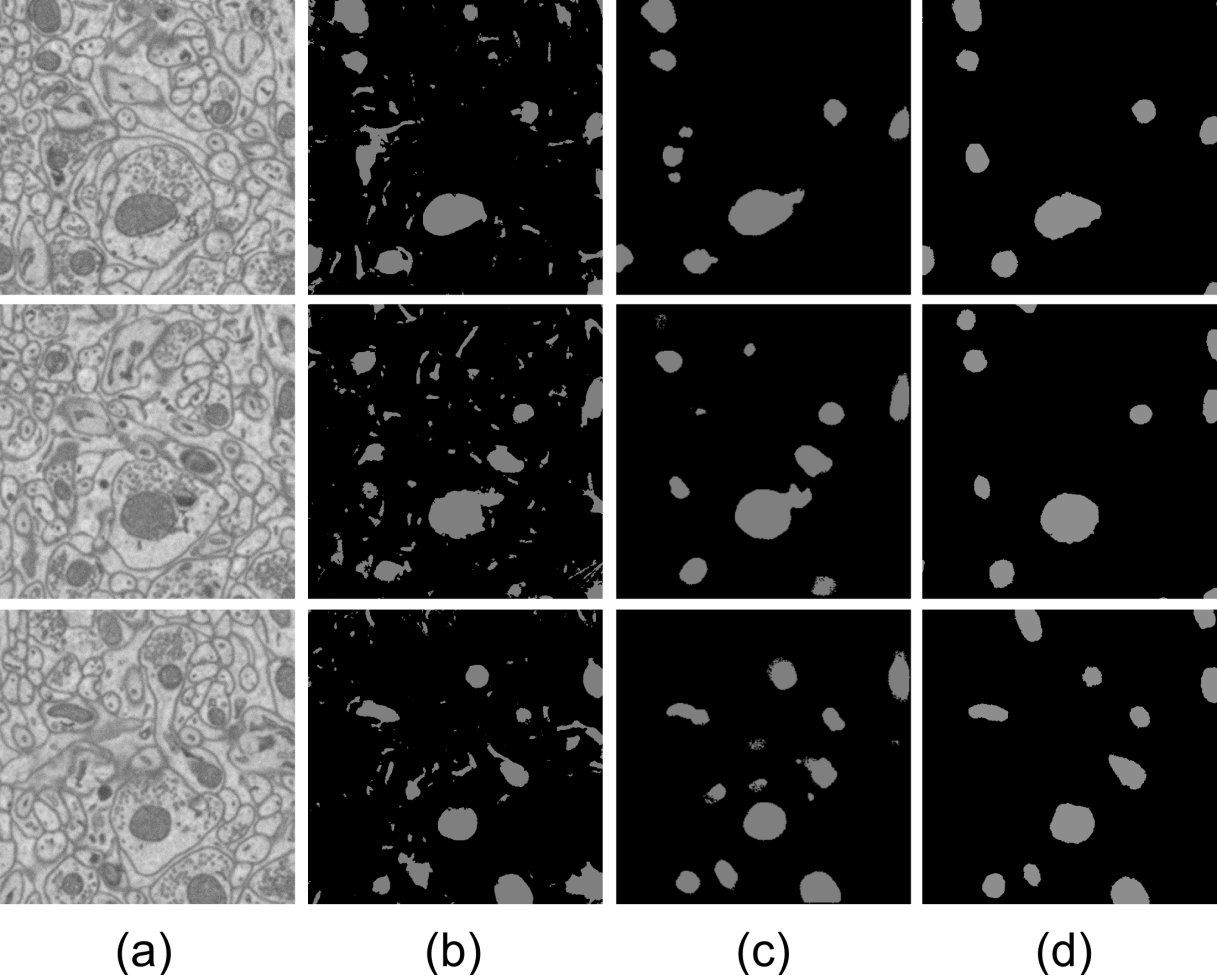
**Automatic results versus manually generated results for Test 1**. Test 1 was performed on cerebellum tissue if a mouse. The XY slices shown here are 3.1 μm × 3.1 μm. Out of the 15 XY slices processed, slice numbers 1, 7, and 13 are shown (from top to bottom). Column (a) shows raw images. Column (b) shows final results of automatic segmentation using Radon-Like Features. Column (c) shows final results of automatic mitochondria segmentation using the CP method presented in this paper. Column (d) shows manually generated mitochondria segmentation. Differences between the automatic (b) and manual (d) images shown here represent error for the process presented in this paper. Differences between the automatic (c) and manual (d) images shown here represent error for the Radon-Like Features method.

**Figure 6:**
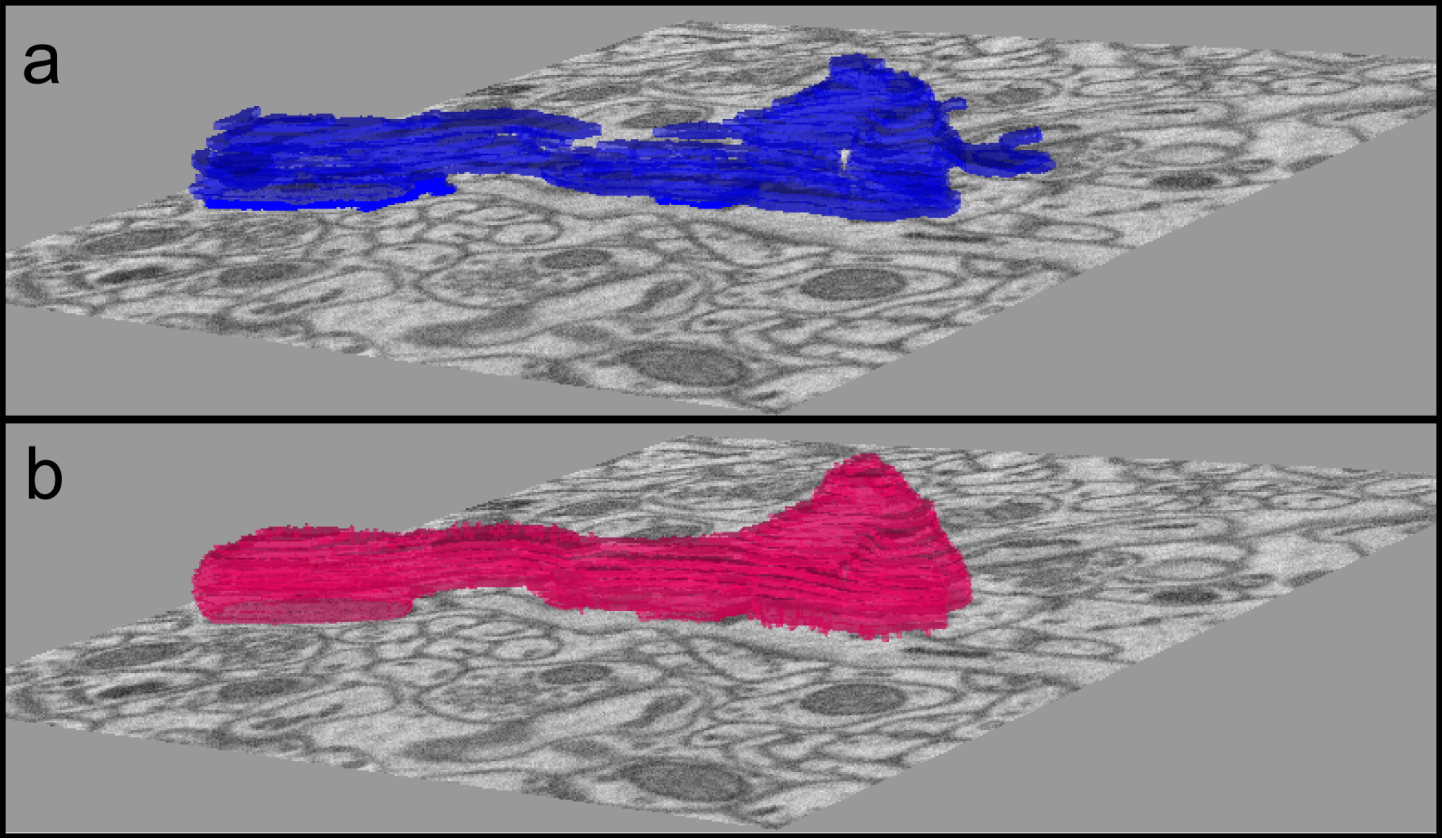
**Smoothing effect of Step 3**. (a) Stack of output contours representing one mitochondria from step 2 using data from Test 1. Contours are rendered as solid slabs. (b) Output of step 3. Notice that the level set operation yields a result that is smoothed. (The slice view for (a) and (b) is 3.5 μm × 3.5 μm in size.)

In step 3, a 3D geodesic active contour level set operation [24] was performed. Results are shown in Figure 5. This operation converts the 2D contour output into smoother 3D objects which are more convenient for viewing and editing than individual contours. The parameters for the operation were set as follows: advection scaling: 160, curvature scaling: 6.75, propagation scaling: 1. The primary purpose of the operation is to increase 3D smoothness, which can simplify the editing process. Smooth output is preferred because jagged edges in XY planes and stair-stepping from slice to slice can be time consuming to correct. Another advantage of smoothing 3D blob output is that manual culling of false positives can be performed on a per 3D blob basis, requiring fewer edits than editing on a per contour basis. In addition, as shown in Figure 3, the level set output has better accuracy that the patch classification alone. Figure 6 shows the smoothing effect of step 3. When applied to the 350 × 350 × 15 block of data referred to in the previous section, the time required for step 3 was 5 minutes.

### Additional testing

Typical use of the system will involve training on a small manually segmented slab from a large acquisition and then applying the automatic segmentation operation to the entire acquisition or chosen portions of it. In addition to Test 1 described above, Tests 2-4 were performed to evaluate the robustness of the method. Test 2 was performed on a different subset of the cerebellum data used in Test 1, using the same training data. Test 3 and Test 4 were performed in different tissues using training sets from corresponding tissue samples. All parameters were the same as with Test 1 unless stated otherwise. Results for all tests are shown in Table 1. The CP method is compared to RLF. The threshold for RLF was chosen in each test so that the true positive rate (TPR) would match the TPR of the CP method. Results indicate that the CP method performs relatively well for each type of data tested.

Setting the threshold value T for each training data set is important. Lower T increases true positives for the complete Cytoseg process but also increases false positives. For each training data set, error measurements were performed to determine an appropriate value for the threshold T. We used only the training data to set this value. To accomplish this, the first 7 slices out of the 15 slices of training data were designated training for the error measurement, and the last 7 slices as test data for the error measurement. The threshold T was set to minimize error E = αf + β(1-t), where f is the false positive rate of the complete Cytoseg process, p is the true positive rate of the complete Cytoseg process, and α and β and terms set by the user to weight the importance of avoiding false positives versus detecting true positives. For all error measurements, we used α = 7 and β = 1. To find the minimum error, the process was run for values of T starting at 0.05 and increasing in increments of 0.05 up to a maximum of 1. For the cerebellum training data used in Tests 1 and 2, T = 0.25. Also for the dentate gyrus training data used in Test 3, T = 0.25. For the CA3 training data, T = 0.1.

Balance of training examples can affect the performance of training. To maintain an approximately consistent ratio of 1:10 for positive example contour pairs to negative example contour pairs, we added n duplicates of each salient contour pair. We used n = 0 for Test 1 and 2, n = 1 for Test 3, and n = 6 for Test 4.

For Test 1, the process was trained on a subvolume of cerebellum as described previously. For patch classification training, 696 positive and 869 negative examples were used. For contour pair classification training, there were 616 positive examples and 5559 negative examples. Results are shown visually in Figure 5.

**Figure 7:**
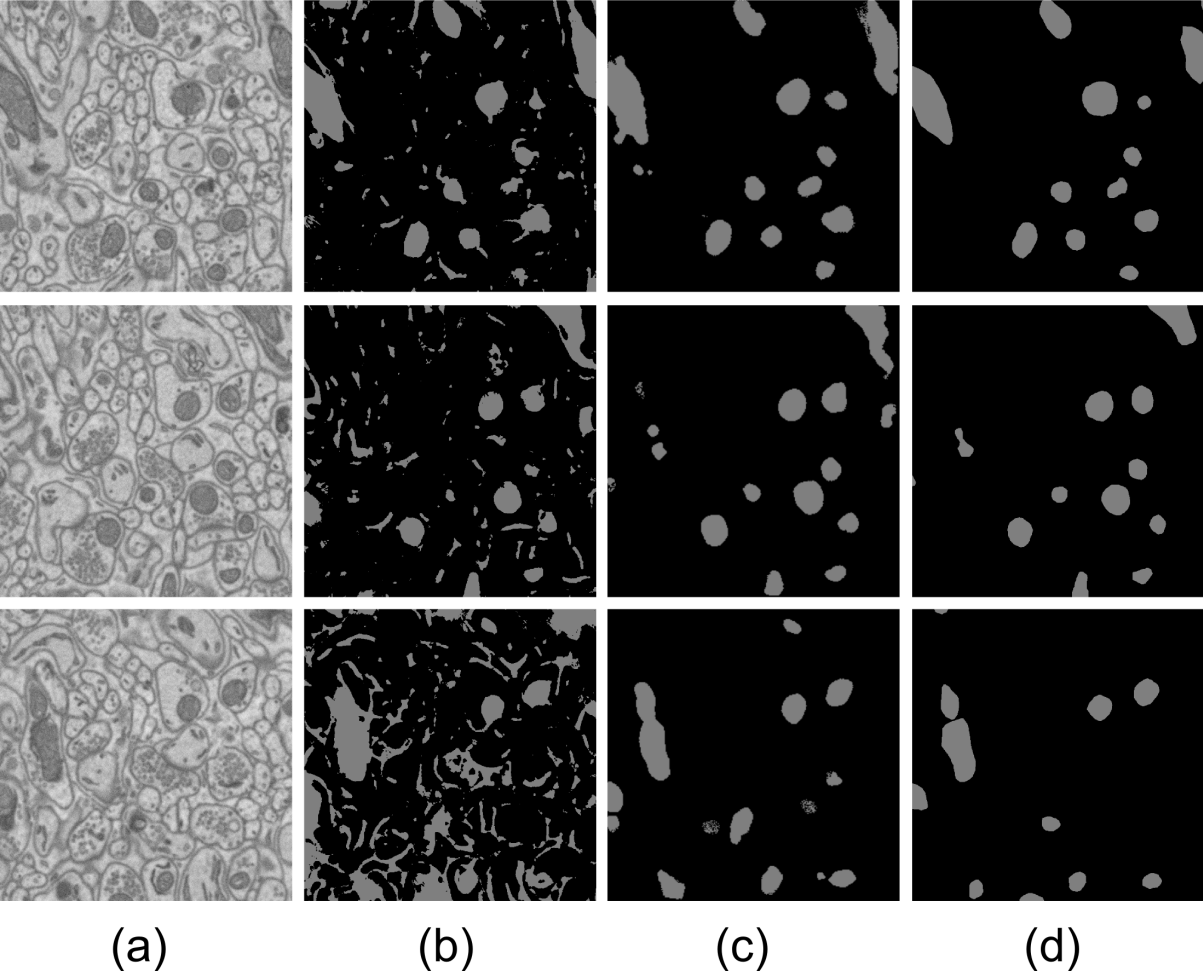
**Automatic results versus manually generated results for Test 2**. Test 2 was performed on a different slab of the cerebellum tissue acquistion used for Test 1. The same training data was used for Tests 1 and 2. The XY slices shown here are 3.1 μm × 3.1 μm. Out of the 15 XY slices processed, slice numbers 1, 7, and 13 are shown (from top to bottom). Column (a) shows raw images. Column (b) shows final results of automatic segmentation using Radon-Like Features. Column (c) shows final results of automatic mitochondria segmentation using the CP method presented in this paper. Column (d) shows manually generated mitochondria segmentation.

For Test 2, our method was applied to a subset of mouse cerebellum different from the subset used in Test 1. This slab of test data was located more than 10 microns away from the slab used for Test 1. The same training data was used as with Test 1. Results are shown in visually Figure 7. Note that the performance is similar within cerebellum for Test 1 and Test 2 (see Table 1).

**Figure 8:**
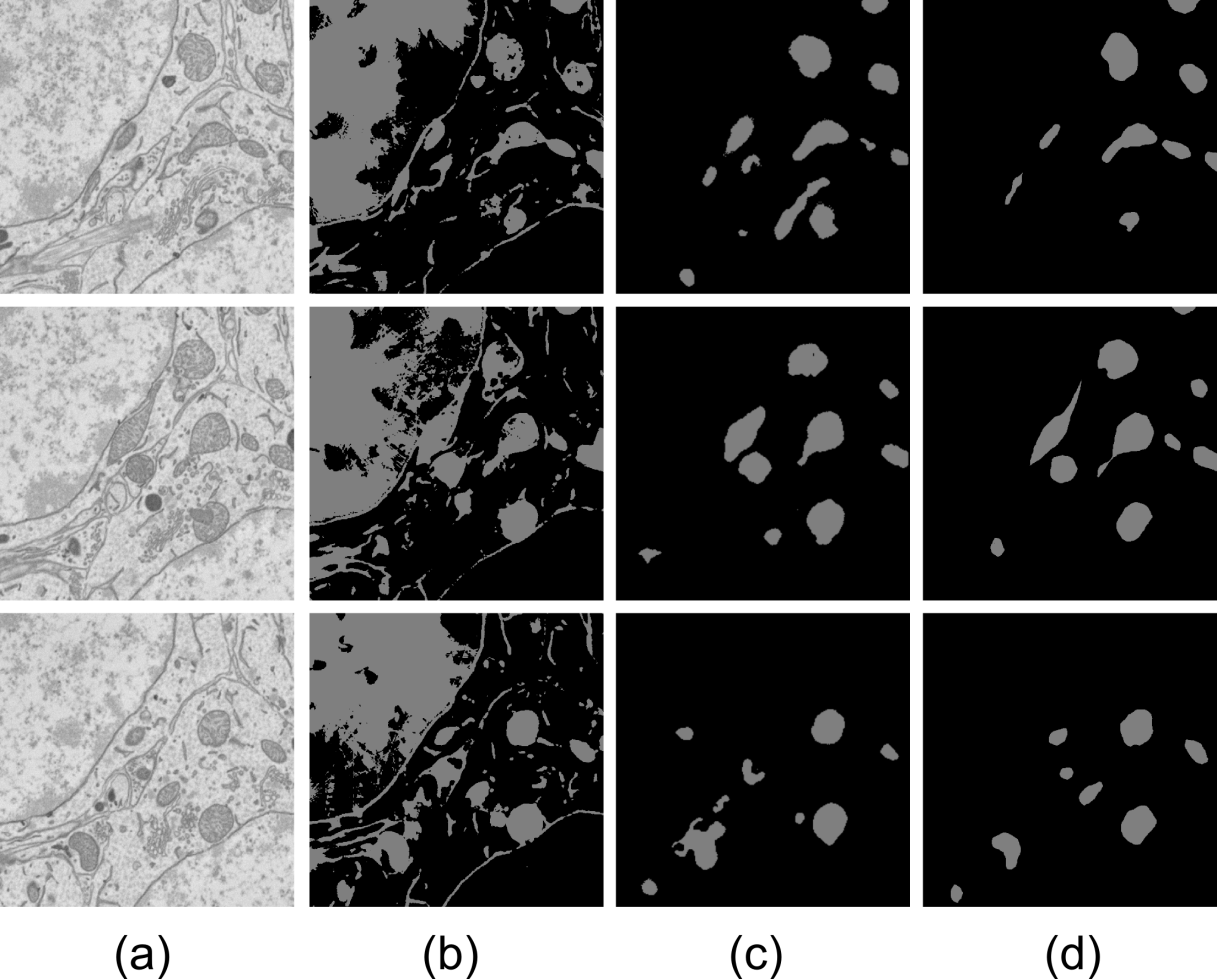
**Automatic results versus manually generated results for Test 3**. Test 3 was performed on a sample of tissue from the dentate gyrus of a mouse. The XY slices shown here are 3.1 μm × 3.1 μm. Out of the 15 XY slices processed, slice numbers 1, 7, and 13 are shown (from top to bottom). Column (a) shows raw images. Column (b) shows final results of automatic segmentation using Radon-Like Features. Column (c) shows final results of automatic mitochondria segmentation using the CP method presented in this paper. Column (d) shows manually generated mitochondria segmentation.

For Test 3, a volumetric image of mouse dentate gyrus tissue data was acquired, and a voxel size of 10 nm × 10 nm × 50 nm was used. For patch classification training, we used 898 positive examples and 959 negative examples. For contour pair classification training, there were 452 positive examples (including duplicates) and 6766 negative examples. Results are shown in Figure 8.

**Figure 9:**
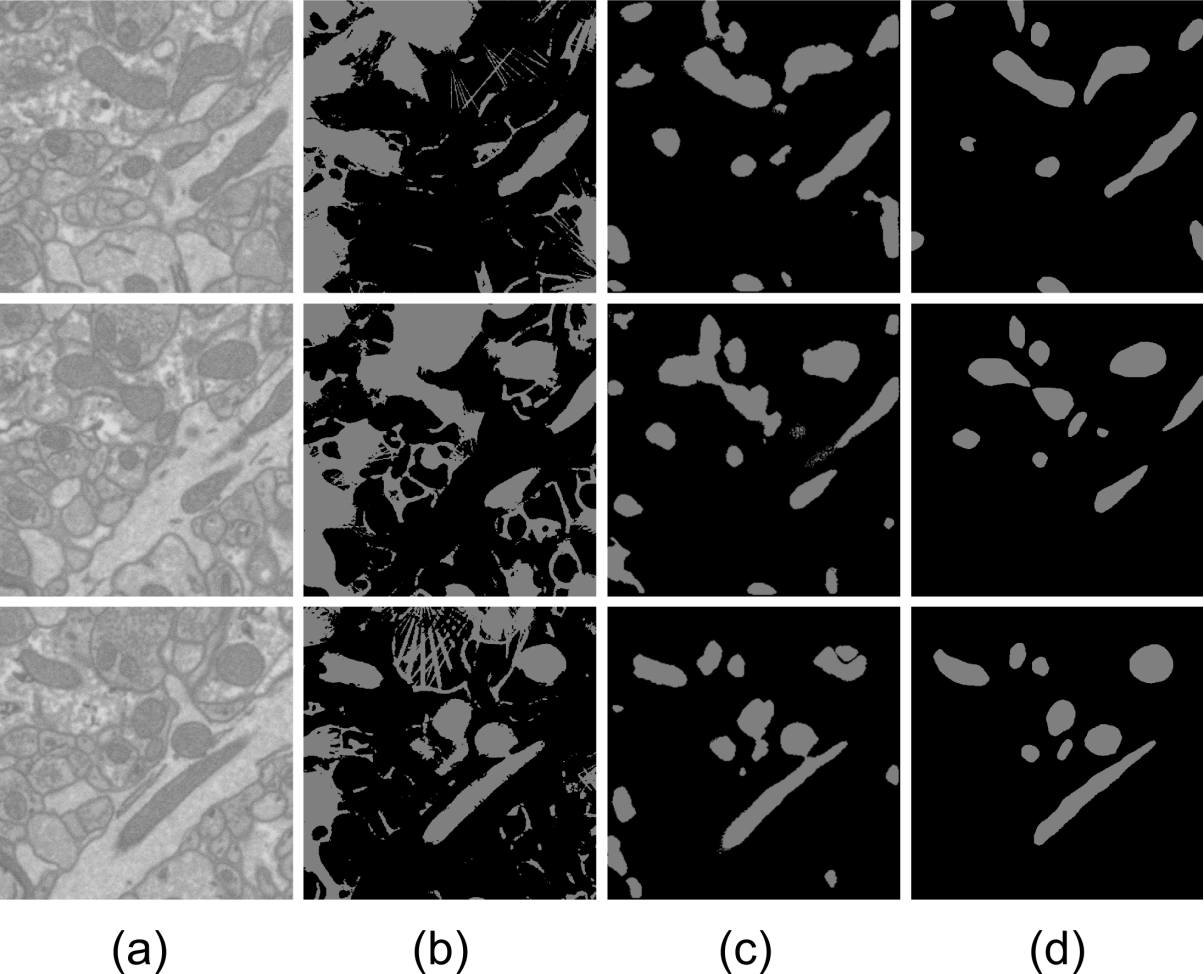
**Automatic results versus manually generated results for Test 4**. Test 4 was performed a sample of tissue from the CA3 region of a mouse hippocampus. The XY slices shown here are 3.1 μm × 3.1 μm. Out of the 15 XY slices processed, slice numbers 1, 7, and 13 are shown (from top to bottom). Column (a) shows raw images. Column (b) shows final results of automatic segmentation using Radon-Like Features. Column (c) shows final results of automatic mitochondria segmentation using the CP method presented in this paper. Column (d) shows manually generated mitochondria segmentation.

For Test 4, a volumetric image of the CA3 division of mouse hippocampus was acquired, and a voxel size of 10 nm × 10 nm × 70 nm was used. For patch classification training, we used 938 positive examples and 934 negative examples. For contour pair classification training, there were 573 positive examples (including duplicates) and 6506 negative examples. Results are shown in Figure 9.

### Implementation

The code for this work was implemented in Python. Python provides a suitable environment for rapid prototyping and has the advantage of being open source and freely available. We utilized ITK and OpenCV libraries as noted previously; both of which have python wrappings. Additionally the Orange [26] data mining library was used at the patch classification stage, and the Python Imaging Library [27] (PIL) was used to load and write images. To ensure reproducibility, our code is available at http://cytoseg.googlecode.com.

To process very large datasets that may exceed random access memory (RAM) limits, the full dataset is programmatically split into multiple slabs of full XY extent but limited Z. Each slab is then processed in the same manner as a full dataset. This provides a simple method to parallelize the process for large datasets, and this or other parallelization methods will be explored in future work.

## Discussion

This work explores the use of multistep segmentation involving patch classification, contour pair classification, and a level set operation. One alternative approach to the segmentation problem is to build a single highly accurate classifier, however this puts a significant burden on the classifier. To handle a complex problem such as automatic segmentation in neuropil, the classifier complexity is typically high, so the training time with a single classifier approach is significant and can require days of CPU time to train [17]. By splitting the problem into stages, training complexity can be reduced at each stage. In our process, the first two steps learn the texture and the shape independently and rapidly. Less than a minute of training time was required for training with the results presented in this paper. While the voxel classification step requires significant time (80 minutes for 2 million patches), it can be distributed among multiple cores as it operates on each patch independently. (Testing on larger datasets using parallel computing resources is a subject of future work.)

We designed each step to add to the accuracy of the process. Our first texture processing step enhances the mitochondria as shown in Figure 2(b), but allows many false positive pixels where texture is similar to mitochondria. The second step uses pairs of contours on adjacent plane and helps to reduce many false positive contour pairs as shown in Figure 2(d). We chose to use contour pairs because single contours are often not sufficient to identify structures. This mimics manual segmentation, where people often check multiple adjacent slices to identify a structure. Use of contour pairs makes use of two planes of information rather than one and increases the accuracy of identification. While the contour pair classification is effective for identifying contours that lie on mitochondria, it often detects somewhat misshapen contours with rough edges. To address this, we introduced a third step. Our third step relies on the fact that mitochondria typically have a smooth shape. A cleaner segmentation can be achieved by enforcing this smoothness using a level set operation.

To fully automate our level set step, we designed it to take seed points from the contour pair classification step and an edge potential map derived from the patch classification step as described earlier. The edge potential map is derived from the patch classification output rather from the raw image so that the level set tends to fill inside of the salient texture. For example, our results for Test 1 show that the final level set operation increased the true positive rate by 0.04 and the false positive rate by 0.0025. Even though the false positive rate increases slightly, the larger true positive rate increase represents an overall improvement. Figure 6 demonstrates this increase in true positives visually as many gaps in the segmentation are filled in with the level set operation.

The resulting combination of all steps is a new pipeline where each contributes to the final result. Compared to a single classifier, this pipeline helps break the problem down into more manageable pieces. In future work, each step can be refined separately, potentially by different image processing specialists, the first focusing on texture identification, the second on shape identification, and the third on enforcing smoothness appropriate for the structure being segmented.

## Conclusions

In this paper, our goal was to develop a method for automatic segmentation of mitochondria in SBFSEM data. These datasets were particularly challenging because many internal structures were stained and spacing between slices was significant. We used both texture and shape information to identify mitochondria in a complex background. The technique involved three major steps that progressively build a final result. Our data had 4 to 7 times more resolution in the XY plane than Z, so we chose a combination of 2D (step 1 and 2) and 3D (step 3) processing that takes advantage of the extra resolution in the XY plane. In the first step, we showed that a random forest classifier applied directly to pixel values of 2D patches has good performance as a patch classifier. In step 2, we showed that detected contours in the probability map from step 1 can be refined using a contour pair classifier. We also showed the advantage of using contour pairs rather than single contours. In step 3, we showed that use of a level set procedure, operating on the probability map from step 1, and seeded with salient contour regions from step 2 can enhance and smooth final results. The output is smoothed in 3D to make visualization and manual corrections more manageable. As show in Table 1, 93-97 percent accuracy is achieved on three types of tissue. In future work, we plan to test with contour sets of three or more to improve contour set classification accuracy.

In addition to accuracy, another advantage of our pipeline design is modularity. As a subject of future work, each of the components of the pipeline may be improved or replaced to suite different problems or improve accuracy. For example, while we used a random-forest based patch classifier to recognize texture, it would be possible to replace this with other texture identifiers.

Although this method was tuned for mitochondria, it is flexible in that texture and shape are learned based on training data. Therefore, if given the proper training data, it may also be applicable to other structures with well defined shape and texture. Application to other structures is a subject for future testing.

## Authors' contributions

RJG designed the segmentation pipeline presented here, implemented the code, tested it against human segmentation, and drafted the manuscript. MEM and MHE participated in the project's coordination and helped to draft the manuscript. All authors read and approved the final manuscript.
